# Endocrine resistant breast cancer: brain metastasis

**DOI:** 10.37349/etat.2022.00081

**Published:** 2022-04-26

**Authors:** Matthew Willman, Jonathan Willman, Brandon Lucke-Wold

**Affiliations:** Department of Neurosurgery, University of Florida, Gainesville, FL 32610-0265, USA; University of Edinburgh, UK

**Keywords:** Endocrine resistant breast cancer, metastasis, brain oncology, emerging therapeutics

## Abstract

Endocrine resistant breast cancer metastasis continues to serve as a significant clinical challenge with high morbidity and mortality for patients. As the number of breast cancer cases continues to rise, the rate of brain metastasis has also increased. For single lesions or a large symptomatic lesion with other smaller lesions, surgical resection is a viable option in non-eloquent regions. Stereotactic radiosurgery is a great option for post-operative therapy or for 10 or fewer small lesions (< 3 cm in size). Whole-brain radiation can be used sparingly for large tumor burdens but should encompass hippocampus sparing techniques. Chemotherapy options have remained relatively limited due to decreased permeability of the blood-brain barrier. Emerging monoclonal antibody treatments have offered initial promise, especially for endocrine resistant breast cancer metastasis.

## Introduction

According to the Centers for Disease Control and Prevention (CDC) 2018 data report published in 2021 254,744 new cases of breast cancer were diagnosed, and 42,465 women died from breast cancer in the United States in a single year [1]. The surveillance epidemiology and end results (SEER) program estimates that in 2021, there were approximately 281,550 new cases of female breast cancer diagnosed and 43,600 deaths in the United States [2]. In 2021, the World Health Organization announced that breast cancer is now more commonly diagnosed than lung cancer, placing breast cancer as the most frequently diagnosed form of cancer worldwide [3]. Breast cancer now accounts for over 1 in every 6 deaths caused by cancer [3]. The majority of breast cancer cases are estrogen receptor (ER) positive (ER+) with their development being heavily reliant on estrogen as a driving factor [4]. While this form of breast cancer is often successfully targeted with endocrine therapies against ER+ receptors, roughly 20% of cases are initially resistant to endocrine therapy, with another 30–40% of cases developing resistance over time [5]. Endocrine resistance can be linked to numerous mechanisms of action, including upregulation of cyclin-dependent kinases (CDKs) 4/6, upregulation of phosphoinositide 3-kinase (PI3K)/AKT and mammalian target of rapamycin (mTOR) cell division signaling pathways, and estrogen receptor alpha (*ESR1*) gene mutation of the tumor ER [6]. Triple-negative breast cancer (TNBC) is a form of endocrine resistant breast cancer that is significant for a lack of targetable receptors ER, progesterone receptors (PR), and human epidermal growth factor receptor 2 (HER2) [7]. The resistant forms of breast cancer, therefore, make hematologic spread to the brain more likely. From a clinical perspective, endocrine resistance is described as either primary or secondary endocrine resistant breast cancer [6]. Primary resistance refers to relapse occurring within 2 years of adjuvant endocrine therapy treatment or progression of disease within 6 months of endocrine therapy for metastatic cancer [8]. Secondary acquired endocrine resistance refers to relapse occurring after 2 years of endocrine therapy treatment or progression of disease after 6 months of endocrine therapy for metastatic cancer [6, 8]. Secondary resistance has been more closely linked to brain metastasis and is a topic of the ongoing investigation.

Due to difficulty treating with targeted therapy, endocrine resistant breast cancer is concerning for high risk of recurrence and high likelihood for brain metastasis. Between 10–30% of breast cancer patients will develop brain metastases [9, 10]. While breast cancer has a generally good prognosis, patients with breast cancer brain metastases (BCBM) typically have a much poorer prognosis with less than 12-month survival [10]. This is especially true for patients with endocrine resistant breast cancer metastasis. According to a 2018 study of 857 patients with BCBM, the most common neurological manifestations leading to referral and ultimate diagnosis of BCBM were headaches, vision changes, focal motor weakness, and focal sensation alterations [9].

**Figure 1.**
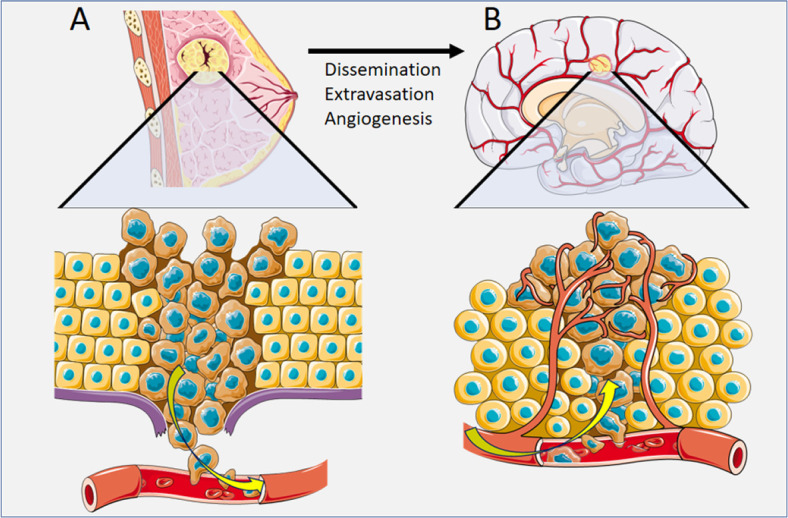
Pathophysiology of cancer spread for breast cancer. (A) Dissemination of breast cancer tumor cells via vascular circulation; (B) Extravasation of breast cancer tumor cells to a secondary site of brain metastasis, with angiogenesis *Note*. This figure contains (modified) images from Servier Medical Art (https://smart.servier.com). CC BY.

Metastasis from a primary tumor requires that the tumor cells enter vascular circulation either by direct contact with blood supply or via lymphatics [11]. Tumor cells are thought to become lodged at branch points of small capillaries within the highly vascularized brain [12]. While a single tumor cell is unlikely to develop into an active metastasis, areas of vasculature that innately accumulate a high burden of tumor cells are believed to interact collectively, promoting the growth of metastasis [13]. The arrested tumor cells initiate a cytokine-mediated pro-inflammatory adhesion to the vascular endothelium, which ultimately leads to tumor cell extravasation through the brain endothelium (Figure 1) [14]. The exact mechanistic pathway of adhesion ligands and receptors varies depending on the unique nature of the primary tumor [14]. Once the tumor cells have extravasated through the capillaries of the brain, the recruitment of supporting vascular nutrient supply is essential for metastatic brain tumor growth [15]. Secretions of growth factors such as vascular endothelial growth factor (VEGF) and brain-derived neurotrophic factor (BDNF) play a key role in the angiogenesis of the supporting vascular network for an active brain metastasis [15].

This review aims to summarize current and future therapeutics and procedures in the field of endocrine therapy-resistant breast cancer brain metastasis.

## Mechanisms of endocrine resistance

Resistance to endocrine therapy can develop due to mutation or loss of endocrine receptors on tumor cells (e.g., hormone receptor-negative and *ESR1* gene mutations) or via the upregulation of pathways independent of endocrine receptors altogether [6, 7, 16, 17].

*ESR1* gene mutations in breast cancer tumor ER are frequently associated with the development of endocrine resistance [16, 17]. Mutations in *ESR1* can cause activation of ER independent of estrogen levels, allowing for the upregulation of breast cancer cell proliferation in an endocrine therapy-resistant mechanism [18]. Mutations causing unregulated activation of *ESR1* present more frequently in metastatic (10–50%) cancer over primary tumors (~1%) [18].

Upregulation of PI3K/AKT and mTOR signaling pathways is highly integrated and is believed to have a significant contribution to the development and maintenance of endocrine resistant breast cancer [19]. PI3K/AKT/mTOR signaling pathway is a kinase pathway that promotes tumor cell proliferation and survival [20]. Additionally, upregulation of this kinase pathway is believed to activate ER via phosphorylation independent of estrogen, allowing for tumor growth in an endocrine resistant process [21]. Promoting cell survival, PI3K/AKT pathway is associated with the inhibition and reduced expression of apoptotic promotors, including procaspase-9 and Fas-ligand [22, 23]. PI3K/AKT pathway is believed to stimulate the activation of the mTOR pathway, which promotes the synthesis of growth factors like VEGF [24, 25]. mTOR-driven VEGF upregulation is a key player in the pro-angiogenetic element of cancer growth [26]. Notably, PI3K/AKT/mTOR upregulation occurs in up to 70% of BCBM, making the PI3K/AKT/mTOR signaling pathway a therapeutic target of interest in BCBM [27].

Forkhead box M1 (FOXM1) is another proto-oncogene that is believed to promote G2 and M phase gene transcription, spindle stability, and mitosis progression [28, 29]. Consequently, studies investigating inhibition of FOXM2 with small interfering RNA (siRNA) have shown increased spindle defects, failure of mitosis, and cancer cell death through apoptosis [30–34]. In addition, there is evidence that in endocrine sensitive breast cancer, the promotion of the ERα pathway may modulate FOXM2 expression and promote proliferation and metastasis to the brain [34, 35]. However, there are numerous estrogen-independent pathways by which FOXM2 may be upregulated, including PI3K/AKT/mTOR and HER2 [35–38]. As a result, FOXM2 is believed to be another key regulator of endocrine resistant breast cancer and may be upregulated in as much as 85% of TNBC cases [30].

CDK4/6 is directly involved in the phosphorylation and subsequent inactivation of tumor suppressor retinoblastoma (Rb) proteins, which ultimately induces cell cycle G1/S transition [39]. This transition promotes DNA synthesis and the progression of the cell towards division. Under normal circumstances, CDK4/6 expression is tightly regulated by endogenous inhibitors [39, 40]. Upregulation of CDK4/6 expression is believed to promote breast cancer tumor cell proliferation in endocrine resistant tumors [40, 41].

Neurofibromin 1 (*NF1*) gene is an important negative regulator of the rat sarcoma virus (Ras)/rapidly accelerated fibrosarcoma (Raf) kinase activation pathway, which is an established promoter of cell proliferation [42, 43]. Mutation or deletion of *NF1* in breast cancer is associated with increased ER phosphorylation, endocrine therapy-resistant breast cancer growth, and ultimately poorer clinical outcome [43]. *NF1* alterations can be present in metastases of invasive lobular carcinoma (mILC) and are typically not seen in metastatic invasive ducal carcinoma (mIDC) [44]. One study of 733 BCBM patients indicated that as many as 9% of BCBM patients had alterations in *NF1* [45].

HER2 upregulation is believed to promote downstream activation of PI3K/AKT pathways, ultimately enhancing tumor cell proliferation and endocrine resistance [19, 25]. Resistance to trastuzumab targeted suppression of HER2 activation in some patients is believed to be partially due to crosstalk between HER2 and Erα [46, 47]. Prolactin receptor (PRLR) and ERα non-genomic signaling is thought to activate HER2 endothelial growth factor pathway, which can, in turn, promote the phosphorylation and activation of ERα via upregulation of PI3K/AKT kinase pathways [48].

## Treatment options

Broad treatment options of BCBM are summarized in Table 1.

**Table 1.** Summary of broad treatment options with general indications and limitations

**Intervention**	**Indication**	**Limitations**
Surgical resection	Single lesion resection and symptom relief	Multiple disseminated lesions
Whole-brain radiotherapy	High metastatic tumor load (> 10 metastases)	Off-target radiation with neurological decline
Stereotactic radiosurgery	Lesion diameters < 3 cm	High metastatic tumor load (> 10 metastases)
Systemic therapy	Therapeutic targets present	Lack of therapeutic targets

### Surgical

Surgical tumor resection is the recommended treatment option for single metastatic tumors that can be accessed without causing significant neurologic deficits [49]. If the tumor is concerning for significant mass effect or obstruction of the ventricular system causing hydrocephalus, surgical resection is generally advised [49]. When determining the scope of the resection, gross-total resection is highly recommended over subtotal resection, as gross-total resection has been shown to comparatively reduce recurrence and improve survival rates [50]. Furthermore, emerging evidence indicates increased survival rates with en bloc resection *versus* piecemeal [50]. In certain cases, a patient with a heavy tumor burden may benefit from surgical resection of a single large tumor causing symptoms for the immediate benefits of symptom relief [51].

### Radiation

While surgical resection of brain metastasis may offer significant benefit of immediate alleviation of symptoms, patients with no adjunct therapies typically experience a recurrence rate of 50–60% at the resection site within 6–12 months postoperative [52, 53]. Postoperative radiation therapy has been shown to significantly reduce the rate of recurrence both at the site of the resection and throughout the brain [52, 53]. Due to studies indicating no overall survival benefit and substantial neurocognitive decline from whole-brain radiotherapy (WBRT), there is an increasing interest in stereotactic radiosurgery (SRS) as the primary adjunct treatment following surgical resection [53]. In situations with heavy metastatic tumor load (> 10 metastases), in which SRS is not advised, WBRT remains the recommended treatment for the management of BCBM [54, 55]. By decreasing the radiation dose to the hippocampal region, hippocampal-avoidance WBRT (HA-WBRT) may significantly reduce memory decline [55].

Single fraction SRS is recommended for the treatment of small tumors with diameters of 3 cm or less. Multiple low-dose hypo-fractionated SRS protocols are being investigated for the treatment of larger tumors (> 2.5 cm) and irregular postoperative margins [54, 56, 57]. Due to the focal nature of SRS therapy and concern for the development of further metastases, regular 2–3 months follow-ups with magnetic resonance imaging (MRI) are recommended by the National Comprehensive Cancer Network guidelines [58].

### Systemic therapy

HER2 is a growth-promoting receptor tyrosine-protein kinase that is frequently elevated in endocrine resistant breast cancer tumors referred to as HER2-positive tumors [59]. HER2+ BCBM may be endocrine resistant and consequently immune to therapies that target hormone receptor-positive breast cancer, such as tamoxifen and fulvestrant. One first-line treatment for HER2+ BCBM is taxane, trastuzumab, and pertuzumab [60, 61]. Trials have indicated that while the monoclonal antibody pertuzumab may have a low blood-brain barrier (BBB) permeability, the concentration in the central nervous system (CNS) is enough to produce a significant therapeutic effect [61, 62].

Trastuzumab emtansine (T-DM1) is often used as a second-line treatment. However, the recent phase 3 trial DESTINY-Breast03 demonstrated a significant improvement of progression-free survival in HER2+ BCBM patients treated with trastuzumab deruxtecan (T-DXd) compared to T-DM1. These results place T-DXd as the mainstay of second-line systemic therapy for HER2+ [63].

Capecitabine and trastuzumab represent another viable therapy. However, a recent study by Lin et al. [64] demonstrated a significant increase in response rate in HER2+ BCBM patients treated with a triple therapy of trastuzumab and capecitabine plus tucatinib compared to trastuzumab and capecitabine alone. In 2020, the Food and Drug Administration (FDA) approved the use of the tyrosine kinase inhibitor neratinib for the treatment of HER2+ BCBM [65]. In addition, the results of the phase 3 Nala trial demonstrated that neratinib with capecitabine therapy significantly increased progression-free survival compared to lapatinib with capecitabine [66]. This suggests that neratinib may be a superior alternative to tucatinib in the aforementioned triple therapy.

Given TNBC’s lack of targetable receptors, including HER, TNBC is a particularly difficult form of BCMB to treat with systemic interventions. Consequently, cytotoxic chemotherapy is the primary systemic therapy used in the treatment of TNBC with BM [57].

### Tumor boards

Conventional tumor boards are multidisciplinary units that usually consist of a variety of medical specialists including radiologists, medical oncologists, nuclear radiologists, pathologists, nurses, palliative care physicians, and surgeons [67]. With the advent of next generation sequencing and drastic reductions in cost and time of sequencing, genetic testing and staging have become integral to the world of cancer therapeutics. Consequently, there has been a push in recent years, especially within academic institutions, to include more research and genetic specialists on tumor board teams forming what is now called molecular tumor boards [68]. Tumor boards often make decisions that significantly change diagnoses, staging, and care plans. One recent study, examining the impact of brain metastasis tumor boards at a large academic hospital, found the tumor board review resulted in significant changes in care for almost 50% of the cases reviewed [69]. However, there has been little research into the impact tumor board decisions may have on patient outcomes such as progression-free survival and quality of life [67, 69].

## Emerging therapeutics

While BBB has microfoci disruption with the presence of breast cancer metastasis, drug permeability for the majority of chemotherapeutic agents remains severely impeded by the remaining intact BBB [70]. Delivery of therapeutic agents through the BBB remains a significant hurdle in the development of new treatments. Recent phase 3 trials indicated that the antibody conjugate sacituzumab govitecan showed a significant improvement of progression-free survival over single-agent chemotherapy for metastatic TNBC [71]. Sacituzumab govitecan may prove a useful therapy in conjunction with other systemic chemotherapy.

As an alternative to SRS, recent research has explored the effectiveness and safety of implantation of low-dose cesium-131 brachytherapy post-resection for recurrence [72]. This may pose a potential benefit for larger resections in which SRS may not be as viable.

**Figure 2.**
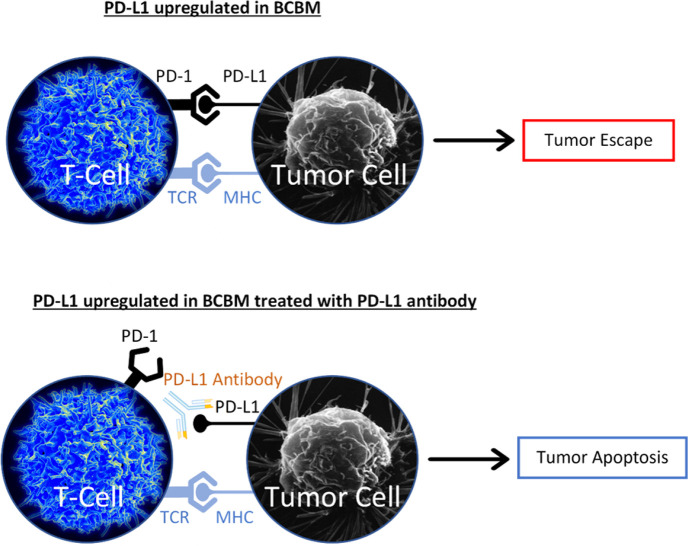
PD-L1 antibody blocks PD-L1 from binding to PD-1. The major histocompatibility complex (MHC) binds to the T-cell receptor (TCR). Without PD-L1/PD-1 interaction, T-cell mediated apoptosis is promoted *Note*. The image representing T-cell is adapted from “Healthy human T cell”, by NIAID (https://www.flickr.com/photos/niaid/5950870236/). CC BY 2.0. The image representing Tumor Cell is adapted from “Breast cancer cell”, by U.S. Department of Health and Human Services: National Cancer Institute (https://visualsonline.cancer.gov/details.cfm?imageid=1989). Public Domain.

Programmed cell death protein 1 (PD-1) receptor ligand (PD-L1) expression has been tied to HER2+ and TNBC brain metastases [73]. PD-L1 is an inhibitory ligand that is responsible for downregulating apoptosis [74]. Recent immunotherapy studies have focused on targeting and inhibiting PD-L1 to promote endocrine resistant BCBM cell apoptosis via an activated T-cell response (Figure 2). One recent trial focuses on SHR-1316, a promising PD-L1 antibody in HER2+ and TNBC brain metastases therapy (NCT04303988). Atezolizumab, another PD-L1 antibody, is currently in a phase 2 trial, examining the drug’s efficacy and safety in conjunction with SRS in TNBC brain metastases (NCT03483012).

CDK4/6 upregulate the phosphorylation of tumor suppressor Rb protein, which plays an integral part in inhibiting the cell cycle pathway progression [6]. When phosphorylated by upregulated CDK4/6, tumor suppressor, Rb protein is inactivated, allowing for the initiation of the cell cycle [75]. Downregulation of CDK4/6 has been shown to promote cell cycle arrest in endocrine resistant breast cancer by inhibiting the activation of DNA syntheses (S) phase of cell division [76]. Abemaciclib, which demonstrates BBB penetration, is being examined for use as an ER+ endocrine resistance metastatic breast cancer treatment to specifically target CDK4/6 and inhibit cell proliferation [6, 77–79].

Given PI3K upregulation prevalence is up to 70% of BCBM and its role in tumorigenesis and endocrine resistance, therapeutic inhibition of PI3K has garnered increased interest in recent years [27]. In 2016, the first-in-human phase 1 trial of paxalisib—a PI3K—demonstrated BBB penetrance [80]. Subsequently, a phase 2 trial examining the use of paxalisib with Trastuzumab for HER2+ patients with BCBM is currently active (NCT03765983). In addition, another phase 2 trial was initiated with a more general scope examining paxalisib and abemaciclib separately in the treatment of brain metastasis after biopsy and genetic testing for viability (NCT03994796). While this study is not specific to BCBM alone, the information it does provide on BCBM may be valuable to the field.

Silibinin inhibits signal transducer and activator of transcription 3 (STAT3), which is expressed by astrocytes that are interacting with tumor cells [81]. STAT3 is believed to promote a pro-metastatic environment for the tumor cells to flourish [81]. Recent experimental animal and human trials have shown encouraging results for STAT3 inhibitors, with a 75% response rate in the initial human trial for BCBM [82]. Due to its limited toxicity, oral bioavailability, and ability to cross the BBB, silibinin may prove to be a promising treatment adjunct for BCBM.

## Conclusions

BCBM management has improved dramatically in the last few decades, with remarkable leaps in anesthesia care, surgery, radiation oncology, and radiology. Unfortunately, the field still has some of the highest mortality rates, as the brain remains a sanctuary site due to its immune-privileged state that is outside the reach of many therapeutic agents.

In addition, endocrine resistant BCBM is one of the most challenging forms of brain metastases to treat with systemic therapy, due to a currently limited number of therapeutic targets. There is a need for research to continue to identify and develop innovative strategies to target the critical pathways of endocrine resistant BCBM growth. Future research will likely look at individually tailored, genetically focused systemic therapeutics, such as antibody therapies that target individual receptors specific to each cancerous tumor. This has strong implications for endocrine resistant breast cancer metastasis.

As cancer care becomes increasingly complicated, and the need for interdisciplinary coordination and communication increases, tumor boards and similar interdisciplinary groups will likely be the future of patient care at larger institutions.

## Abbreviations

BBB: blood-brain barrier
BCBM: breast cancer brain metastases
CDKs: cyclin-dependent kinases
ER: estrogen receptor
ER+: estrogen receptor positive
*ESR1*: estrogen receptor alpha
FOXM1: forkhead box M1
HER2: human epidermal growth factor receptor 2
mTOR: mammalian target of rapamycin
*NF1*: neurofibromin 1
PD-1: programmed cell death protein 1
PD-L1: programmed cell death protein 1 receptor ligand
PI3K: phosphoinositide 3-kinase
Rb: retinoblastoma
SRS: stereotactic radiosurgery
STAT3: signal transducer and activator of transcription 3
TNBC: triple-negative breast cancer
VEGF: vascular endothelial growth factor
WBRT: whole-brain radiotherapy

## Declarations

### Copyright

© The Author(s) 2022.
